# Proximal Femoral Replacement for Prosthetic Hip Joint Infection

**DOI:** 10.7759/cureus.95512

**Published:** 2025-10-27

**Authors:** Sathya Lakpriya, Lucy Maling, Gihan Jayasinghe, Richard Slack

**Affiliations:** 1 Department of Trauma and Orthopaedics, East Kent Hospitals University NHS Foundation Trust, Kent, GBR

**Keywords:** femoral stem, hip infection, infected hip arthroplasty, pfr, pji, prosthetic hip joint infection, prosthetic joint infection, proximal femoral replacement, revision hip arthroplasty, revision hip surgery

## Abstract

Introduction: Revision total hip arthroplasty is a surgical challenge when undertaken for hip prosthetic joint infection (PJI). When PJI occurs in the setting of severe proximal femoral bone loss, proximal femoral replacement (PFR) can serve as an effective salvage strategy, restoring function in prosthetic hips that cannot be reconstructed otherwise.

Materials and methods: This retrospective series from a high-volume revision hip surgeon comprises 15 PFRs in 14 patients. Infection was the indication for surgery in all cases. Thirteen patients had confirmed PJI as per the 2018 Musculoskeletal Infection Society (MSIS) criteria. The primary outcome measure was the eradication of infection. Secondary outcome measures included peri- and postoperative complications, re-operation, mobility, and death. The mean follow-up was three years.

Results: Infection was eradicated in 13 cases (86.6%). Of the two cases with infection, one was asymptomatic for seven years before PJI became evident again. Complications included hip dislocation (three episodes in two patients). One patient underwent revision of the acetabular component due to infected loosening. All but one patient were ambulatory with walking aids. Both patients with persistent or recurrent infection subsequently died.

Conclusions: PFR is an option for patients with PJI for whom standard revision hip arthroplasty would be unsuitable and excision surgery unacceptable. This study suggests promising results for the eradication of PJI using PFR as a salvage procedure in this particularly challenging cohort.

Level of evidence: The study is classified as level IV evidence.

## Introduction

Revision total hip arthroplasty is an exponentially increasing burden. This rise coincides with an aging population and an increase in the number of primary surgeries (92,874 in 2018 compared to 67,491 in 2008 across England, Wales, and Northern Ireland) [1]. The risk of prosthetic joint infection (PJI) following primary total hip arthroplasty is quoted as 1-2% [2,3] and may be as high as 14% in revision surgery, as reported by Bozic et al. [4]. Infection is an important indication for revision; the National Joint Registry (NJR) lists infection as the indication for 17% of revisions in 2018, compared to 14% in 2010 [1].

The economic burden of PJI is considerable. The procedural cost of hip revision surgery for PJI in the United Kingdom exceeds £21,000 per patient, compared to £12,000 for aseptic loosening [5]. Additionally, one must consider the individual costs to the patient, including pain, immobility, repeated operations, and even death. Various surgical strategies exist for the management of hip PJI, from debridement and retention of implants (DAIR) to complete component exchange [6]. Component exchange surgery may be performed as a single-stage or a two-stage procedure, with the latter considered the gold standard [7-9].

Proximal femoral replacement (PFR) was initially developed for the reconstruction of large bony defects associated with oncological orthopedic surgery [10]. It has gained popularity in the non-oncological setting for conditions associated with significant bone loss, such as PJI [11,12].

## Materials and methods

This retrospective case series reports on 15 PFRs performed in 14 patients with confirmed or suspected PJI. All procedures were carried out by a high-volume revision hip surgeon at Queen Elizabeth The Queen Mother (QEQM) Hospital in Margate, Kent, a university teaching hospital within the East Kent Hospitals University NHS Foundation Trust. Inclusion criteria were PFR performed specifically for confirmed or suspected PJI and a minimum postoperative follow-up of six months. The single exclusion criterion was PFR performed for any other indication.

**Figure 1:**
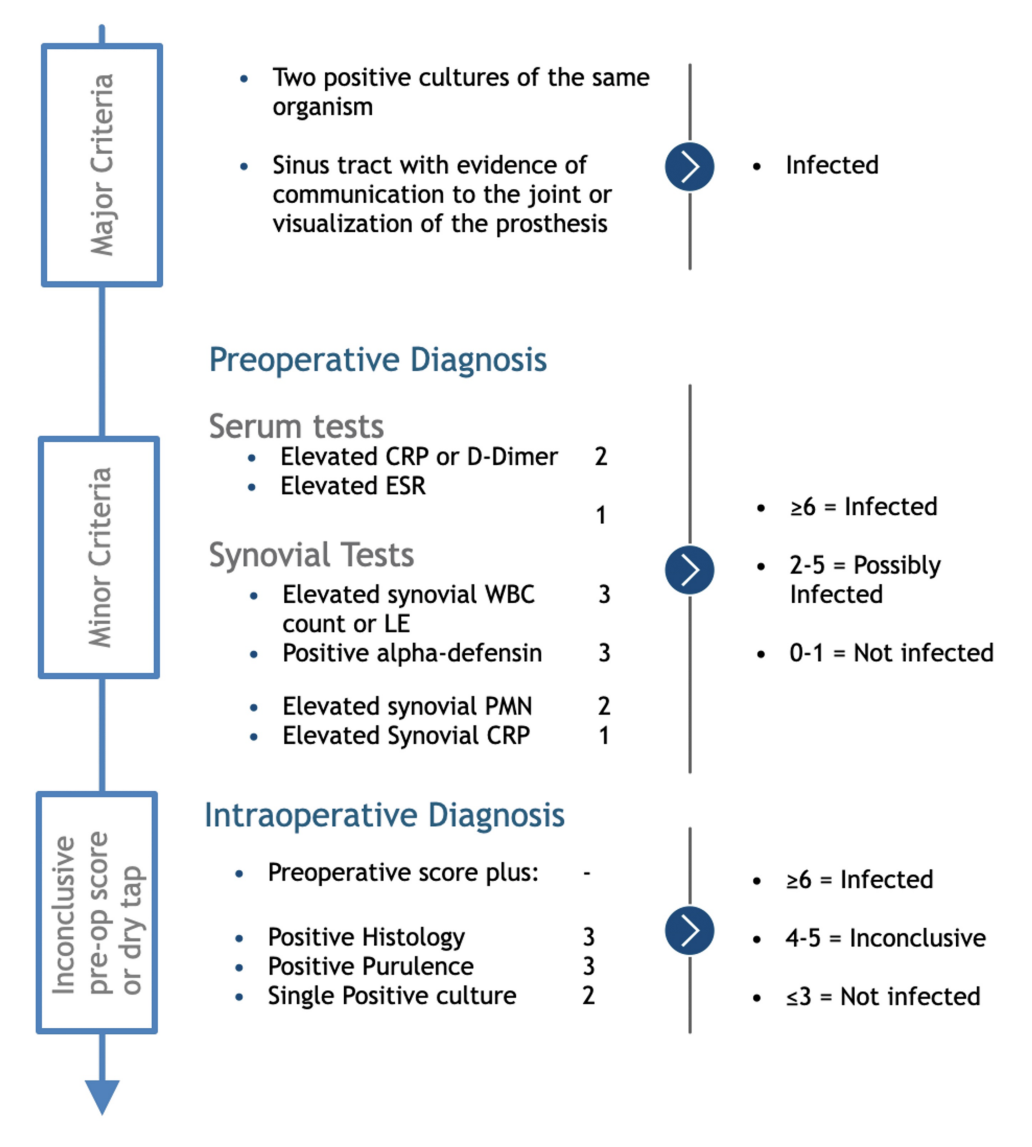
2018 Musculoskeletal Infection Society Criteria for prosthetic joint infection CRP: C-reactive protein; ESR: erythrocyte sedimentation rate; WBC: white blood cell; PMN: polymorphonuclear neutrophils; LE: leukocytes; pre-op: preoperative Reprinted from Journal of Arthroplasty, Vol. 33, Parvizi J, Tan TL, Goswami K, Higuera C, Della Valle C, Chen AF, Shohat N, “The 2018 definition of periprosthetic hip and knee infection: an evidence-based and validated criteria,” pp. 1309-1314, Copyright (2018), with permission from Elsevier [13].

Data were collected from electronic medical records, including operative notes, outpatient clinic letters, laboratory investigations, and radiographic imaging. Variables recorded included patient demographics, number and type of previous surgeries, American Society of Anesthesiologists (ASA) Physical Status Classification System grade, comorbidities, and microbiological and histopathological results. The diagnosis of PJI was made in accordance with the 2018 Musculoskeletal Infection Society (MSIS) criteria [13], as depicted in Figure 1. These criteria include major indicators such as the presence of a sinus tract communicating with the prosthesis and isolation of the same pathogen from two separate tissue or fluid samples, as well as minor criteria involving serological markers and intraoperative findings.

The primary outcome measure was eradication of infection, defined as the absence of clinical signs of inflammation, in conjunction with C-reactive protein (CRP) and erythrocyte sedimentation rate (ESR) values of less than 5 mg/L and 30 mm/h, respectively [14]. Secondary outcome measures included peri- and postoperative complications, re-operation for any reason, postoperative mobility, and mortality.

The study cohort consisted of 10 female and four male patients, with a mean age of 77 years (range: 63-92 years). The average follow-up duration was three years (range: six months to nine years). Patients had undergone a mode of three prior hip procedures (range: 0-4), including first-stage revisions, and all had at least two medical comorbidities. Most patients were classified as ASA grade 3.

PFR was performed as a salvage procedure in lieu of excision arthroplasty. Ten of the 15 cases were conducted as part of a two-stage revision procedure, with the initial stage involving explantation of existing components and radical debridement. The remaining five procedures, including two cases presenting with periprosthetic fracture, were performed as single-stage interventions. One single-stage case involved a patient with a pathological femoral fracture secondary to *Mycobacterium tuberculosis* infection of the native hip.

All surgeries were performed via a posterior approach. Radical debridement and excision of diseased tissue were guided by defined tissue planes, employing techniques adapted from orthopedic oncology. PFR was achieved using either the Biomet® Orthopedic Salvage System (Zimmer Biomet, Warsaw, USA) or the Stryker® Global Modular Replacement System (Stryker Orthopedics, Portage, USA). Intraoperative goals included restoration of anatomical version, offset, leg length, and soft tissue tension. Reconstruction of the abductor mechanism was attempted where feasible. All patients were managed postoperatively in a multidisciplinary framework involving orthopedic surgeons, microbiologists, anesthetists, and physiotherapists. Antibiotic regimens were tailored according to intraoperative cultures and microbiological guidance. Postoperative care included early mobilization with full weight-bearing, while observing standard hip precautions.

## Results

Ten of the 15 cases satisfied the 2018 MSIS major criteria for the diagnosis of PJI. This was defined by the presence of a sinus tract communicating with the prosthesis and/or a pathogen isolated by culture from two separate tissue or fluid samples. Ten had positive cultures, and a sinus was present in three. One patient had bilateral prosthetic hip infection with the same pathogen. The most common pathogen cultured was *Staphylococcus aureus*, which was present in over half of the positive cultures; one of these was a methicillin-resistant strain. Also cultured were *Klebsiella*, *Pseudomonas aeruginosa*, group B streptococcus, *Staphylococcus haemolyticus*, and *Staphylococcus cohnii*.

Five of the 15 cases had neither a positive culture nor a communicating sinus. PJI was diagnosed in three of these patients using the 2018 MSIS minor criteria and/or intraoperative findings. Two patients did not meet the 2018 MSIS criteria for diagnosis and were considered 'inconclusive'; data were lacking for synovial fluid analysis. Both had intraoperative documentation of suspected infection. One such patient had a history of *Mycobacterium tuberculosis* infection of the hip joint in youth. This patient presented with a pathological fracture of the proximal femur against a background of suspected infection. Histology results supported this diagnosis.

Primary outcome measure

The primary outcome measure was the eradication of infection. Patients were considered infection-free if they met all of the following criteria: absence of clinical signs of inflammation, serum values for CRP and ESR of <5 mg/L and <30 mm/h, respectively. Thirteen of the 15 cases (86.6%) were clear of infection at their most recent follow-up.

The other two patients both had gram-negative diagnoses. One had a persistently draining sinus, which was managed with long-term suppressive antibiotics, and died eight months later. One patient had PJI confirmed after a seven-year asymptomatic period following PFR. This patient re-presented with symptomatic loosening of the acetabular component, along with contralateral hip PJI. The same pathogen was isolated during acetabular revision on the PFR side. This patient died the following year after a period of suppressive antibiotic therapy.

Secondary outcome measures

Hip dislocation occurred in three cases (20%). One hip was dislocated twice, requiring open reduction each time. This patient underwent an acetabular revision for septic loosening, as described above. All dislocations occurred within four months of the index procedure or revision. Perioperative medical complications included pulmonary embolus (1) and lower respiratory tract infection (1). Both patients recovered with medical management.

The most recent postoperative radiographs were reviewed for evidence of loosening. The DeLee and Charnley acetabular zones, as well as the remaining Gruen femoral zones, were assessed. The mean radiographic follow-up was 3.3 years (range: six months to nine years). One patient had evidence of loosening of both the acetabular and femoral cement-bone interfaces at six-year follow-up; this patient subsequently had an acetabular revision for infective loosening. Asymptomatic heterotopic ossification was noted in three patients at one-year follow-up. No other patients had radiographic evidence of loosening.

All but one patient were ambulatory with mobility aids following surgery. Six used one or two sticks, six used a roller frame, two used one or two crutches, and one was wheelchair-bound. All returned to their previous place of residence. Both patients with persistent or recurrent infection subsequently died. Another two patients died from unrelated causes.

## Discussion

This case series belongs to a high-volume revision hip surgeon who performs over 100 hip revisions per year, many of which are for PJI. PFR comprises a very small proportion of this operative workload and is reserved for those whose treatment requires a radical approach. These patients have often exhausted standard arthroplasty options and have multiple poor predictive factors, including virulent organisms, poor bone stock and soft tissues, frailty, and comorbidity. It cannot be overemphasized that this is a salvage procedure.

PFR is a revision solution for the challenging situation posed by PJI in the presence of poor bone stock. Unlike conventional revision surgery that utilizes impaction cementation or allograft composites, it does not rely on the integrity of the proximal femur, nor does it require union at the host bone-allograft site. Crucially, patients are allowed to weight bear immediately after surgery. This is a clear advantage compared to excision arthroplasty, which may lead to long-term immobility.

PFR is gaining popularity, but there continues to be sparse literature surrounding its use for non-oncological reasons. Very few studies have examined its use in the management of prosthetic hip joint infection. A recent systematic review found infection eradication rates of 91.8% and 92.1% for conventional single and two-stage revision hip arthroplasties, respectively [15]. Dieckmann et al. used a two-stage revision with PFR in 49 patients with PJI, noting an equivalent 92% cure rate [16]. However, in a systematic review of 61 PFR procedures for PJI, Korim et al. quoted a lower cure rate of 78.9% [17]. These authors postulated that those undergoing PFR were likely to have had a higher number of previous surgeries and may have had more resistant organisms.

Our results showed a cure rate of 86.6%, which is higher than that noted by Korim et al. but similar to that found by Dieckmann et al. In fact, this figure may be an underestimate: one patient was symptom-free for seven years after PFR before the infection became apparent again. This may have represented a new rather than persistent infection, particularly as she underwent contralateral hip surgery for PJI shortly before re-presenting, which may have been a potential source. Nevertheless, this case was counted as 'persistent infection' in our results for consistency.

Dieckmann et al. noted dislocation to be the most common complication, occurring in 12% of cases [16]. High rates of dislocation have also been found in PFR performed for non-infective, non-neoplastic indications [18-20]. The typical patient undergoing PFR is elderly, comorbid, and has had multiple surgical insults to the soft tissues. It may not be surprising that dislocation is a considerable problem in this cohort. To date, there is little evidence regarding surgical approach, the use of bi- or tripolar bearings, or recommended head size to reduce dislocation risk in PFR. Repair of the residual abductor mechanism is generally recommended [12,18,20]. Intensive tailored perioperative physiotherapy as part of the multidisciplinary management may help to avoid dislocation.

Aseptic loosening was not a problem in this case series. Radiographic evidence of infective loosening occurred in one patient with persistent or recurrent infection. Three patients were noted to have asymptomatic heterotopic ossification. Where possible, the remaining periosteal sleeve was closed around the proximal femoral implant intraoperatively. This may have given rise to new bone formation surrounding the prosthesis, as seen on postoperative radiographs.

Limitations

The main limitation of this study was the sample size. PFR is considered a salvage option for PJI and is typically reserved for patients who have exhausted alternative conventional revision surgery options. It was impossible to draw statistical conclusions with this sample size.

Furthermore, missing data rendered two patients’ diagnoses of infection 'inconclusive'. Synovial alpha-defensin testing was not available in this hospital trust due to cost. Additionally, extended duration cultures and synovial fluid counts were not routinely performed. It is important to note that many of these patients had multiple courses of antimicrobial treatment, which may have led to further diagnostic uncertainty. Improved diagnostics may be achieved if patients are managed by a specialist multidisciplinary team that includes microbiologists and histopathologists with a special interest in bone infection.

Another limitation of this study was the variation in follow-up duration. As previously described, this procedure is often performed on elderly and comorbid patients. It is not surprising that some patients may be lost to follow-up due to death, cognitive decline, or relocation. However, we believe the early postoperative period is important to investigate, as complications tend to occur during this time.

This study used eradication of infection as the primary outcome measure. Functional outcome measures may help to support or refute the use of PFR for PJI, but were not obtained in this study. Al-Taki et al. found statistically significant improvements in the quality of life for patients with PFR compared to those who underwent conventional revision total hip arthroplasty for both infective and non-infective indications [21]. Further research with a larger sample size is required to evaluate the medium- and long-term outcomes in patients with PFR.

## Conclusions

PJI poses many challenges similar to bone tumors, which are managed by specialist oncological orthopedic surgeons in the setting of an expert multidisciplinary team. The management of PJI requires a similar approach and should include a high-volume revision surgeon. PFR is an option for patients with PJI for whom standard revision hip arthroplasty would be unsuitable and excision surgery unacceptable. This study suggests promising results for the eradication of PJI using PFR in this particularly challenging cohort.
